# Comparison of pars plana with anterior chamber glaucoma drainage device implantation for glaucoma: a meta-analysis

**DOI:** 10.1186/s12886-018-0896-x

**Published:** 2018-08-29

**Authors:** Bin Wang, Wenwei Li

**Affiliations:** Department of Ophthalmology, Tongde Hospital of Zhejiang Province, 234 Gucui Road, Hangzhou, 310012 China

**Keywords:** Glaucoma drainage device, Pars plana, Anterior chamber, Meta analysis

## Abstract

**Background:**

The purpose of this study was to compare the efficacy and safety of pars plana glaucoma drainage device (PP GDD) with anterior chamber glaucoma drainage device (AC GDD) for the treatment of glaucoma.

**Methods:**

We comprehensively searched three databases, including PubMed, EMBASE, and the Cochrane Library databases, selecting the relevant studies. The continuous variables, namely, intraocular pressure (IOP) and glaucoma medications, were pooled by the weighted mean differences (WMDs), and the dichotomous outcomes, including corneal failure incidence and overall complications incidence, were pooled by the odds ratio (ORs).

**Results:**

Four retrospective studies involving 275 eyes were evaluated, with 135 in the PP GDD group and 140 in the AC GDD group. The WMDs of the IOP reduction between the PP GDD group and the AC GDD group were − 1.01 mmHg (95% CI -4.05 to 2.03, *p* = 0.52). The WMDs of the glaucoma medications reduction between the PP GDD group and the AC GDD group were 0.23 (95% CI -0.11 to 0.56, *p* = 0.19). The pooled ORs comparing PP GDD group with AC GDD group were 1.01 (95% CI 0.03 to 40.76, *p* = 0.99) for corneal failure incidence and 1.19 (95% CI 0.68 to 2.09, *p* = 0.54) for overall complication incidence. There were no significant differences between PP GDD group and AC GDD group on these aspects.

**Conclusions:**

Both PP GDD and AC GDD procedures had similar efficacy of reduction in the IOP and number of medications. They are also both comparable on the safety with similar incidence of corneal failure and overall complications.

**Electronic supplementary material:**

The online version of this article (10.1186/s12886-018-0896-x) contains supplementary material, which is available to authorized users.

## Background

Glaucoma drainage device (GDD) was invented by Molteno in 1969 [1]. Since then, it has been widely used in the management of refractory glaucoma for more than 4 decades. A high success rate with an excellent intraocular pressure (IOP) control is achieved. In the last few years, some surgeons even recommended it as a first-line surgery for glaucoma.

In a standard procedure, the GDD is applied in the anterior chamber (AC) and serves as a shunter to draw aqueous humor through a tube to a subconjunctival end plate. However, it has been reported that many serious complications occurred during the follow up, especially in the anterior segment, such as corneal failure, flat anterior chamber, hyphema etc. [2–7]. Lots of attempts have been made to address these problems. This, in turn, usually aggravates the burden of patients both physically and mentally. Besides, for patients with inadequate anatomical space in the AC or those with previously compromised corneas, it is not feasible.

As an alternative surgery, pars plana (PP) insertion of a GDD into the vitreous cavity was first described in 1991 [8]. Nevertheless, it has its own operative risks as well, including vitreous incarceration of the tube, vitreous hemorrhage, and retinal detachment [9].

Until recently, only a few comparative studies have compared these two kinds of surgeries in the treatment of glaucoma [10–16]. To the best of our knowledge, comparisons of the efficiency and safety between these two methods have not been systematically reviewed and published. Therefore, we systematically analyzed the available literature to evaluate the efficiency and safety of PP GDD implantation versus AC GDD implantation for glaucoma.

## Methods

### Literature search

A comprehensive literature search of PubMed, EMBASE, and Cochrane library was performed to identify the relevant studies by two independent reviewers. The search methodology and search keywords are presented in Additional file 1. The search strategy used both keywords and Medical Subject Headings (MeSH) terms. No language or date restrictions were applied. The computerized searches covered the period from inception to April 2018.The full-text articles were retrieved for the manuscripts that potentially matched the inclusion criteria.

### Inclusion and exclusion criteria

The inclusion criteria for eligibility were as follows: (1) comparisons of the efficacy and/or safety between PP GDD implantation and AC GDD implantation for glaucoma were reported; (2) prospective and retrospective comparative controlled clinical studies were included, because of the paucity of randomized controlled trials (RCTs) on PP GDD implantation and AC GDD implantation; and (3) the inclusion of at least one of the outcomes of interest. Exclusion criteria were as follows: (1) abstracts from conferences and full texts without raw data available for retrieval; (2) duplicate publications, letters, and reviews. For publications reporting on the same study, the most informative and recent article was included in this analysis. Slight disagreements between the reviewers were resolved by consensus.

### Data extraction

Data were extracted from each included study by 2 independent reviewers. Slight discrepancies between the 2 independent data extractions were resolved by the discussion. For the eligible studies, the following data were extracted: (1) study characteristics, including the first author, year of publication, country, study design, number of eyes involved, patient demographics and follow-up time; (2) efficacy outcomes, including preoperative and postoperative IOP, preoperative and postoperative glaucoma medications; (3) safety outcomes, including the incidence of corneal failure and overall complications, such as tube obstruction, tube/plate exposure, device removal, retinal detachment, vitreous hemorrhage, hyphema, choroidal effusion, flat anterior chamber, corneal failure, cystoid macular edema, strabismus, diplopia, hypotony, loss of light perception, etc. Since other outcomes including visual field progression were not specifically recorded in the studies, we did not analyze these aspects.

### Quality assessment

The methodological quality of each study was assessed based on the Newcastle-Ottawa Scale for quality of case–control studies in meta-analysis; for this assessment, and we used the Newcastle-Ottawa Scale star system (range, 0 to 9 stars) [17]. Two reviewers subjectively reviewed all studies and assessed all the aspects that influence the quality of a study, including selection, comparability and exposure.

### Outcome measures

The primary outcome was the IOP reduction from preoperative to the last follow-up. The secondary outcome measure was the difference in the reduction in glaucoma medications from preoperative to the last follow-up. The outcomes of safety were complication rates in either group, including corneal failure and overall complications described before during the operation and whole follow-up time.

### Statistical analysis

Data analysis was performed using Review Manager 5 software (RevMan 5, The Cochrane Collaboration, Oxford, UK). For continuous outcomes, the mean and SD were used to calculate weighted mean differences (WMDs). For dichotomous outcomes, odds ratios (ORs) were calculated. Statistical heterogeneity among studies was evaluated with the x^2^ and I^2^ tests [18]. *p* < 0.05 was considered statistically significant on the test for overall effect.

## Results

### Literature search

**Fig. 1:**
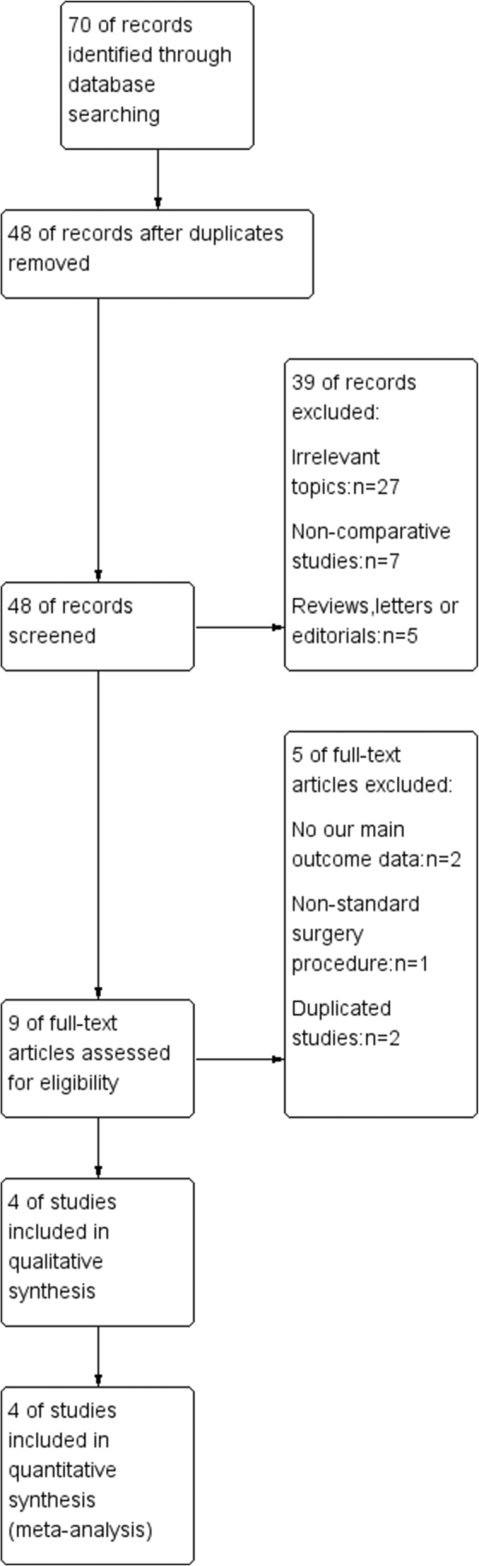
Flow chart of study identification

The selection of studies is shown in Fig. 1. A total of 70 articles were initially identified. 48 studies were left for further analysis after duplications removed. The abstracts were reviewed, and the remaining 9 studies were retrieved for a full-text review. Finally, 4 studies [10–12, 14] that enrolled a total of 275 eyes (135 in the PP GDD group and 140 in the AC GDD group) were included in this analysis.

### Study characteristics and quality assessment

The main characteristics of the four included studies is listed in Table 1. These studies were conducted in several countries, as was shown in Table 1. In total, 275 eyes were enrolled, with 135 in the PP GDD group and 140 in the AC GDD group. Their mean age ranged from 52 to 67.3 years. All of them were retrospective studies. The average follow-up period varied from 18.0 months to 43.5 months. The qualitative assessment of these studies is summarized in Table 2.

**Table 1:** Study characteristics of eligible clinical studies

Study	Year	Country	Study Design	Gender(Female:Male)		Ethnicity(Black:White:Asian:other)		Assignments		No. of eyes		Mean Age(years)		Follow-up time(months)	
				Pars Plana	Anterior Chamber	Pars Plana	Anterior Chamber	Pars Plana	Anterior Chamber	Pars Plana	Anterior Chamber	Pars Plana	Anterior Chamber	Pars Plana	Anterior Chamber
Maris	2013	USA	R	19:12	18:13	2:17:1:11	3:10:3:15	PK,PAS,AACA	NA	31	31	67.3	65.7	20.9 ± 12.4	20.5 ± 13.1
Qin	2018	USA	R	30:27	36:21	20:35:2:0	23:32:1:1	LCEC,FD,CT	NA	57	57	63.1 ± 19.6	64.1 ± 20.5	43.5 ± 24.8	35.3 ± 16.7
Rososinski	2015	Australia	R	11:17	17:16	NA	NA	CP,CG,PV	NA	29	34	59	52	28	18
Seo	2015	Korea	R	9:9	10:8	NA	NA	NA	NA	18	18	52.9 ± 15.7	53.4 ± 17.8	18.0 (15 ∼ 20)	18.0 (16 ∼ 20)

*PK* penetrating keratoplasty, *PAS* peripheral anterior synechiae, *AACA* abnormal anterior chamber angle, *LCEC* low corneal endothelial count, *FD* Fuchs’ dystrophy, *CT* corneal transplant, *CP* corneal pathology, *CG* corneal graft, *PV* previous vitrectom, *NA* not applicable

**Table 2:** Newcastle-Ottawa Scale table

Study	Selection	Comparability	Measurement	Total
Case-control studies				
Maris 2013	3	2	2	7
Qin 2018	3	1	2	6
Rososinski 2015	2	0	2	4
Seo 2015	3	1	2	6

### Efficacy analysis

Since the time of follow-up in all the included studies lasted 1.5 years or more, the outcomes were believed to stabilize and may not make a difference on the statistical analysis. Therefore, the outcomes were analyzed from pre-operation to the final follow-up.

#### Intraocular pressure reduction

**Fig. 2:**
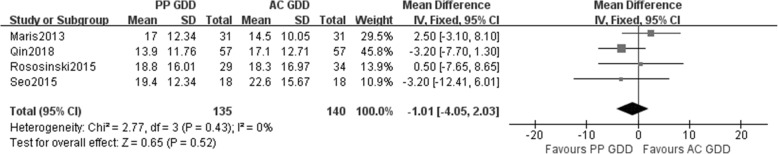
Reduction of IOP between PP GDD group and AC GDD group

All the included studies reported preoperative and postoperative IOP. They demonstrated that the mean IOP reduction was similar in both groups, and the meta-analysis of pooled data did not show any statistically significant differences between the two groups (mean difference = − 1.01 mmHg, 95% CI -4.05 to 2.03, *p* = 0.52) (Fig. 2).

#### Glaucoma medications reduction

**Fig. 3:**
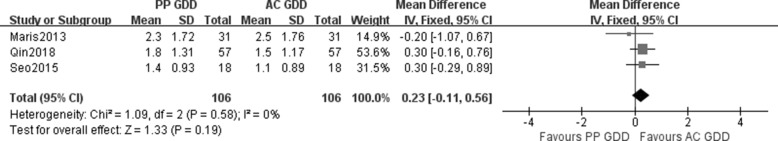
Reduction of glaucoma medications between PP GDD group and AC GDD group

There were 3 outcomes illustrated in the 3 studies as to the glaucoma medications reduction. Examination of the forest plot showed that the differences between the two groups were not significantly different (mean difference = 0.23, 95% CI -0.11 to 0.56, *p* = 0.19) (Fig. 3).

### Safety analysis

#### Corneal failure

**Fig. 4:**
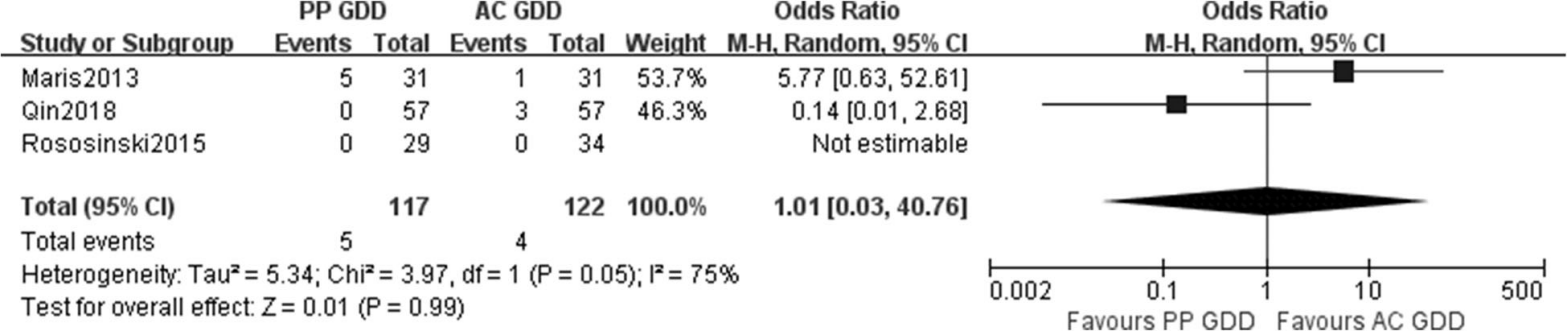
Incidence of corneal failure between PP GDD group and AC GDD group

Three studies reported data for the corneal failure incidence. However, examination of the forest plots revealed that the differences were not statistically significant between the two groups (mean difference = 1.01, 95% CI 0.03 to 40.76, *p* = 0.99) (Fig. 4).

#### Overall complications

**Fig. 5:**
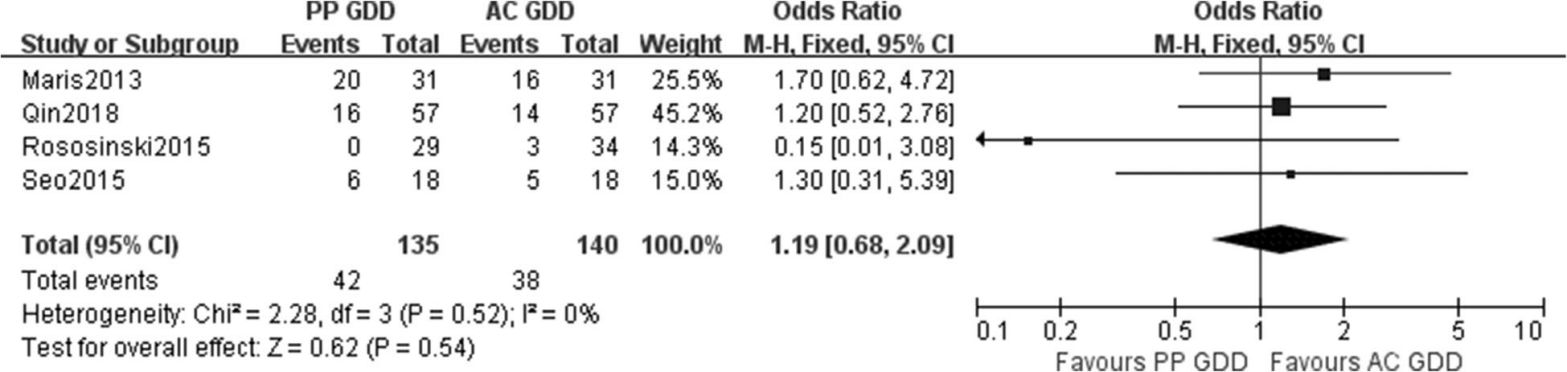
Incidence of overall complications between PP GDD group and AC GDD group

Overall complications comparing the two groups were described in all of the included studies. And these were displayed in Table 3. Pooled results showed similar overall complication incidence between the 2 groups (mean difference = 1.19, 95% CI 0.68 to 2.09, *p* = 0.54) (Fig. 5).

**Table 3:** All the complications included in the studies

Complications	Maris		Qin		Rososinski		Seo	
	PP(31)	AC(31)	PP(57)	AC(57)	PP(29)	AC(34)	PP(18)	AC(18)
Tube obstruction	4	2	NA	NA	NA	NA	NA	NA
Tube/plate exposure	2	0	NA	NA	NA	NA	NA	NA
Device removal	1	0	NA	NA	NA	NA	NA	NA
Retinal detachment	1	1	NA	NA	0	1	NA	NA
Vitreous hemorrhage	1	0	1	3	NA	NA	2	1
Hyphema	1	0	NA	NA	NA	NA	1	3
Choroidal effusion	3	4	NA	NA	NA	NA	NA	NA
Flat anterior chamber	0	6	NA	NA	NA	NA	NA	NA
Cystoid macular edema	1	1	NA	NA	NA	NA	NA	NA
Strabismus	1	0	NA	NA	NA	NA	NA	NA
Corneal failure	5	1	NA	NA	NA	NA	NA	NA
Loss of light perception	0	1	NA	NA	NA	NA	NA	NA
Diplopia	NA	NA	9	3	NA	NA		
Hypotony	NA	NA	5	5	NA	NA	1	0
Tube erosion	NA	NA	1	0	NA	NA		
Retinal hemorrhage	NA	NA	NA	NA	0	1	NA	NA
Tube replacement	NA	NA	NA	NA	0	1	NA	NA
Elevated IOP	NA	NA	NA	NA	NA	NA	2	1

*NA* not applicable

## Discussion

In our present study, four retrospective clinical studies were reviewed. After pooling the results of these studies, we found that both procedures shared similar efficacy of reduction in the IOP and number of glaucoma medications. For safety, both procedures resulted in similar complications in terms of corneal failure and overall complications.

In a long time, glaucoma drainage devices have been reserved for patients diagnosed with refractory glaucoma. However, with its favorable advantages, it has been introduced to routine surgical management of glaucoma patients as well. In the traditional surgery, GDD is inserted into the anterior chamber. And it shunts aqueous outflow from the anterior chamber into the subconjunctival space, which could lower IOP to normal values. In spite of all these advantages, the routine placement of GDD has been associated with serious anterior segment complications [19]. To avoid this, pars plana insertion of a GDD into the vitreous cavity was first described in 1991 [8]. Theoretically, the posterior location of GDD could reduce the risk of anterior segment complications. And in recent years, several clinical studies were designed to compare the therapeutic effects between the two methods [10–12, 14]. According to what we know, this is the first meta analysis to evaluate clinical effects and safety between PP GDD and AC GDD for glaucoma.

In our analysis, we found that the reductions of IOP and glaucoma medications in both groups were both remarkable. According to the four included studies, there was no statistically significant difference in efficiency between two procedures.

On the other hand, the incidence of corneal failure and overall complications were compared and analyzed respectively. Since PP GDD avoided the interference of anterior chamber, it was conjectured that it might come out with a lower corneal failure incidence. However, based on our analysis, we did not find any obviously difference. Of these, only in one study, Seo et al., 2015 compared the changes in corneal endothelial cells after PP GDD with those after AC GDD and its average follow-up was 18 months [12]. It showed significant difference between the 2 groups, which illustrated that endothelial cell damage in the PP GDD group appeared to be lower than that in the AC GDD group. Other clinical studies did not show specific details on endothelial cell number. Some further studies may be needed to confirm the effect on endothelial cell. So far, the two procedures had similar corneal failure incidence. This may be explained by the reason that the effect on corneal endothelial cells may not be so obviously different that the incidence of corneal failure was unable to detect this change in both groups. With regard to overall complications, the two groups did not show any significant advantages over the other one. This may be explained by the fact that clinicians have more effective means to intervene in case of anterior segment complications and the outcomes are better. However, when it comes to the posterior segment complications, the surgical intervention and treatment are limited with poor outcomes generally. Several studies analyzed this and our results were similar to theris [10–16].

However, several limitations should be taken into account when considering the results of this meta analysis. First, in reviewing the literature, the studies included are all retrospective studies because of the absence of randomized studies in the database, which may have potential sources of selection bias. There is still the possibility of underlying bias where there are different clinical indications for placing AC and PP GDD. As the PP is often reserved for cases where AC is not feasible or the cornea is at a high risk, we cannot exclude this bias. This needs to be confirmed with further RCTs, which are still rare in the publishing studies. Typically for meta-analysis research, publication bias cannot be excluded. Additionally, the pooled data were only from the mean follow up of various studies of different durations, introducing a potential heterogeneity. And we speculated that outcomes were stabilized after 1.5 years. Later, the state of IOP, number of glaucoma medications and the incidence of corneal failure and overall complications became stable. The changes were subtle if the differences existed. On the other hand, different studies adopted different criteria for participants. It might be another source of heterogeneity in the results. Also, the antiglaucoma therapy difference among the studies served as another point of heterogeneity, which should not be neglected. Finally, because of the limited number of studies available in the analysis, we did not perform subgroup analysis.

## Conclusions

Our results showed that both the PP GDD and AC GDD procedures had similar efficacy of reduction in the IOP and number of medications. They are also both comparable on the safety with similar incidence of corneal failure and overall complications. However, there is still an urgent need for pragmatic RCT with long duration and a large sample size to further determine the efficacy and safety (especially, the endothelial cell number change) of PP GDD in the treatment of glaucoma.

## Additional file

Additional file 1: Search strategy. (DOCX 12 kb)

## Abbreviations

AC: anterior chamber
CI: Confidence interval
GDD: Glaucoma drainage device
IOP: Intraocular pressure
OR: Odds ratio
PP: Pars plana
R: Retrospective
RCT: Randomized controlled clinical trial
WMD: Weighted mean difference
