# Supplementary material for: Comparison of pars plana with anterior chamber glaucoma drainage device implantation for glaucoma: a meta-analysis
Source: BMC Ophthalmol. 2018 Aug 29;18:212. doi: 10.1186/s12886-018-0896-x (PMC6114491; doi:10.1186/s12886-018-0896-x)
Supplement: Supplementary file 1 — Search strategy. (DOCX 12 kb) [file 12886_2018_896_MOESM1_ESM.docx]

Search strategy

PUBMED:

(((((((("Glaucoma Drainage Implants"[Mesh]) OR (((((((((((((((((((((((Drainage Implant, Glaucoma[Title/Abstract]) OR Drainage Implants, Glaucoma[Title/Abstract]) OR Glaucoma Drainage Implant[Title/Abstract]) OR Implant, Glaucoma Drainage[Title/Abstract]) OR Implants, Glaucoma Drainage[Title/Abstract]) OR Aqueous Shunts[Title/Abstract]) OR Aqueous Shunt[Title/Abstract]) OR Shunt, Aqueous[Title/Abstract]) OR Shunts, Aqueous[Title/Abstract]) OR Glaucoma Filtration Implants[Title/Abstract]) OR Filtration Implant, Glaucoma[Title/Abstract]) OR Filtration Implants, Glaucoma[Title/Abstract]) OR Glaucoma Filtration Implant[Title/Abstract]) OR Implant, Glaucoma Filtration[Title/Abstract]) OR Implants, Glaucoma Filtration[Title/Abstract]) OR Aqueous Humor Shunts[Title/Abstract]) OR Aqueous Humor Shunt[Title/Abstract]) OR Shunt, Aqueous Humor[Title/Abstract]) OR Shunts, Aqueous Humor[Title/Abstract]) OR Baerveldt Implants[Title/Abstract]) OR Implants, Baerveldt[Title/Abstract]) OR Krupin Valves[Title/Abstract]) OR Valves, Krupin[Title/Abstract]))) AND (("Anterior Chamber"[Mesh]) OR (((Anterior Chambers[Title/Abstract]) OR Chamber, Anterior[Title/Abstract]) OR Chambers, Anterior[Title/Abstract]))))))) AND pars plana[Title/Abstract]
